# Deregulation of IL-1β axis in peripheral blood mononuclear cells from patients with Chronic Recurrent Multifocal Osteomyelitis

**DOI:** 10.1186/1546-0096-9-S1-P307

**Published:** 2011-09-14

**Authors:** Roberta Scianaro, Antonella Insalaco, Giusi Prencipe, Luisa Bracci-Laudiero, Anna Maria Teti, Fabrizio De Benedetti

**Affiliations:** 1 IRCCS Ospedale Pediatrico Bambino Gesù, Rome, Italy; 2 Istituto di Farmacologia Traslazionale, CNR, Rome, Italy; 3 Dipartimento di Medicina Sperimentale, Università dell’Aquila, Italy

## Background

Chronic recurrent multifocal osteomyelitis (CRMO) is a rare childhood disease presenting with bone pain, fever and multifocal osteolytic bone lesions. CRMO is considered an autoinflammatory disease but its aetiology and pathogenesis are still unknown. Autoinflammatory diseases are usually characterized by abnormalities in the regulation of inflammasomes, macromolecular complexes involved in the processing and in the release of IL-1β.

## Aim

To investigate the inflammasome response in CRMO patients.

## Methods

In this study, 10 CRMO patients (mean age 10.7 ± 5.4 years) and 9 age-matched controls were enrolled. The basal mRNA expression of inflammatory cytokines such as IL-1β, IL-6 and TNF-α and of inflammasome components such as ASC, NLRP3 and CASP-1 were evaluated by quantitative real-time PCR in peripheral blood mononuclear cells (PBMCs). IL-1β levels released in conditioned media after LPS and ATP treatment of PBMCs were measured by ELISA.

## Results

mRNA levels of IL-1β and IL-6 were significantly higher in PBMCs from CRMO patients than in healthy donors, with a ten-fold average increase in IL1β transcript (p= 0.0001) and a five-fold average increase of IL-6 transcript (p=0.001). mRNA levels of inflammasome components were comparable between the two groups. When PBMCs were stimulated in vitro with LPS alone, we observed higher levels of IL-1β in the conditioned media of PBMCs obtained from CRMO patients than in those obtained from controls (p=0.01). Controls cells released high IL1β levels only after stimulation with LPS and ATP with no difference with PBMCs from CRMO patients.

## Conclusion

Our data suggest that an abnormal regulation of IL-1β axis characterize PBMCs of CRMO patients.

